# Icotrokinra induces early and sustained pharmacodynamic responses in phase IIb study of patients with moderate-to-severe psoriasis

**DOI:** 10.1172/jci.insight.193563

**Published:** 2025-12-22

**Authors:** David Strawn, James G. Krueger, Robert Bissonnette, Kilian Eyerich, Laura K. Ferris, Amy S. Paller, Andreas Pinter, Dylan Richards, Elizabeth Y. Chen, Kate Paget, Daniel Horowitz, Roohid Parast, Joshua J. Rusbuldt, Jocelyn Sendecki, Sunita Bhagat, Lynn P. Tomsho, Ching-Heng Chou, Marta E. Polak, Brice E. Keyes, Emily Bozenhardt, Yuan Xiong, Wangda Zhou, Cynthia DeKlotz, Paul Newbold, Dawn M. Waterworth, Megan Miller, Takayuki Ota, Ya-Wen Yang, Monica W.L. Leung, Lloyd S. Miller, Carolyn A. Cuff, Bradford McRae, Darren Ruane, Arun K. Kannan

**Affiliations:** 1 Johnson & Johnson, Spring House, Pennsylvania, USA.; 2 The Rockefeller University, New York, New York, USA.; 3 Innovaderm Research, Montreal, Quebec, Canada.; 4 University of Freiburg, Freiburg im Breisgau, Germany.; 5 University of North Carolina, Chapel Hill, North Carolina, USA.; 6 Northwestern University Feinberg School of Medicine and the Ann & Robert H Lurie Children’s Hospital, Chicago, Illinois, USA.; 7 Goethe University Frankfurt, Frankfurt, Germany.; 8 Johnson & Johnson, La Jolla, California, USA.; 9 Johnson & Johnson, Horsham, Pennsylvania, USA.; 10 Johnson & Johnson, Cambridge, Massachusetts, USA.

**Keywords:** Clinical Research, Dermatology, Inflammation, Biomarkers, Clinical trials, Transcriptomics

## Abstract

**BACKGROUND:**

Icotrokinra is the first and only targeted oral peptide that selectively binds the IL-23 receptor with high affinity to precisely inhibit IL-23 signaling. Icotrokinra demonstrated high rates of complete skin clearance and durable disease control in the phase IIb trial, FRONTIER-1, and its long-term extension, FRONTIER-2, in participants with moderate-to-severe plaque psoriasis. This study evaluated systemic and skin pharmacodynamic response of icotrokinra and its relationship to clinical response in FRONTIER participants.

**METHODS:**

FRONTIER-1 participants received icotrokinra or placebo for 16 weeks. FRONTIER-2 followed participants for up to 1 year of treatment; placebo participants transitioned to icotrokinra after week 16. Systemic pharmacodynamic changes were assessed in serum through week 52. Skin pharmacodynamic changes were assessed using transcriptomic analysis of skin biopsies and protein quantification in tape-strip samples through week 16.

**RESULTS:**

Icotrokinra dose-dependently reduced serum levels of the IL-23/IL-17 axis and psoriasis disease biomarkers through week 52, with maximal reductions observed with the highest 100 mg twice-daily dose. Proteomic analyses showed icotrokinra selectively blocked IL-23–driven inflammation without broader impacts on circulating proteins, including serum IL-23 levels. Sixteen weeks of icotrokinra, but not placebo, reduced expression of psoriasis-associated genes in lesional skin. Icotrokinra treatment also reduced psoriasis-relevant proteins in week 16 lesional skin tape-strips to levels comparable to nonlesional samples.

**CONCLUSION:**

Icotrokinra induced a dose-dependent pharmacodynamic response, with early (week 4) and sustained (week 52) reductions in biomarkers of IL-23 pathway activation and psoriasis disease severity, which correlated with clinical response.

**TRIAL REGISTRATION:**

ClinicalTrials.gov: NCT05223868, NCT05364554.

**FUNDING:**

Johnson & Johnson.

## Abstract 2

Icotrokinra, a targeted oral peptide that selectively blocks IL-23R activation, induced early and durable pharmacodynamic responses in participants with moderate-to-severe plaque psoriasis driving disease improvement.

## Introduction

Psoriasis is a common, chronic, immune-mediated, inflammatory disease affecting around 2%–4% of the population in Western countries (1), with plaque psoriasis accounting for about 90% of cases (2). The pathogenesis of psoriasis involves a complex interplay between T cells, innate immune cells, and keratinocytes (3). Cytokines and other inflammatory mediators secreted by immune cells drive leukocyte infiltration and keratinocyte proliferation (3). This cutaneous inflammation results in the clinical appearance of psoriatic skin lesions that are histologically characterized by loss of keratinocyte terminal differentiation, epidermal hyperproliferation, and thickening (3).

Conventional systemic oral therapies, including methotrexate, cyclosporine, and fumaric acid esters, have limited efficacy and may be associated with serious side effects (4). Despite the availability of multiple advanced systemic oral and biologic treatment options, a significant portion of eligible patients with moderate-to-severe plaque psoriasis are not on advanced therapy, due to safety concerns, lower efficacy of advanced oral therapies, and/or patient preference (5). Biologic therapies targeting several cytokine pathways, including tumor necrosis factor (TNF), IL-12/23, IL-17, and IL-23 signaling, have been approved for treating psoriasis and generally deliver greater efficacy but are limited by the need for intravenous or subcutaneous injections (6, 7). Two advanced oral therapies, apremilast (phosphodiesterase-4 inhibitor) and deucravacitinib (tyrosine kinase 2 inhibitor), have been recently approved for psoriasis but show lower efficacy or safety/tolerability concerns relative to biologics, limiting their use (8–10). Thus, for patients with psoriasis, there is a significant unmet need for an oral therapy with higher efficacy and a more favorable safety and tolerability profile.

The IL-23 receptor (IL-23R) signaling pathway plays a critical role in the pathogenesis of psoriasis. Genetic studies have shown that polymorphisms in the IL-23 pathway are associated with an increased risk of developing psoriasis (11, 12). IL-23 is predominantly secreted by myeloid cells and can activate IL-23R^+^ pathogenic T cells (T helper 17, T cytotoxic 17, and tissue-resident memory T cells) and innate immune cells (mucosal-associated invariant T cells, innate lymphoid cells, γδ T cells, and natural killer cells) to drive skin inflammation (13–17). Furthermore, evidence supports a role for IL-23R signaling in differentiating pathogenic versus nonpathogenic cells across autoimmune diseases (18). IL-23 pathway inhibition with monoclonal antibodies targeting the p19 subunit of IL-23 or the p40 subunit shared by IL-12 and IL-23 is clinically validated and has high therapeutic efficacy in psoriasis (19–24). However, the IL-23R has not previously been a therapeutic target for psoriasis, and there are no approved, oral therapies that selectively target the IL-23 pathway. Icotrokinra (previously JNJ-77242113) is the first and only targeted oral peptide that selectively binds IL-23R with high affinity to precisely inhibit proximal IL-23R signaling and downstream cytokine production (25). Preclinical studies found icotrokinra to be a stable and bioavailable oral peptide with no risk of drug-drug interaction (26). Unlike currently approved IL-23 inhibitors that target the soluble IL-23 ligand (e.g., guselkumab, risankizumab, and tildrakizumab), icotrokinra is a competitive antagonist of the IL-23R and blocks IL-23 signaling, differentiating this compound from currently approved advanced therapies in psoriasis.

In the recently completed phase IIb, randomized, double-blind, placebo-controlled, dose-ranging, 16-week study (FRONTIER-1, NCT05223868) in participants with moderate-to-severe psoriasis, icotrokinra showed a significant dose-response relationship (*P* < 0.001) (27). In this study 79% of participants receiving 100 mg twice daily achieved at least 75% reduction in the Psoriasis Area and Severity Index score (PASI 75), and 40% achieved complete skin clearance (PASI 100) at week 16 (27). In the 36-week long-term extension (LTE) study (FRONTIER-2, NCT05364554), no new adverse events or safety signals were identified, and clinical responses were sustained through 52 weeks of treatment (28). Here, modulation of systemic and tissue pharmacodynamic (PD) biomarkers and its relationship to clinical response was evaluated to develop a deeper understanding of targeting IL-23R for therapeutic benefit in psoriasis.

## Results

### Participant baseline clinical and molecular characteristics.

**Figure 1:**
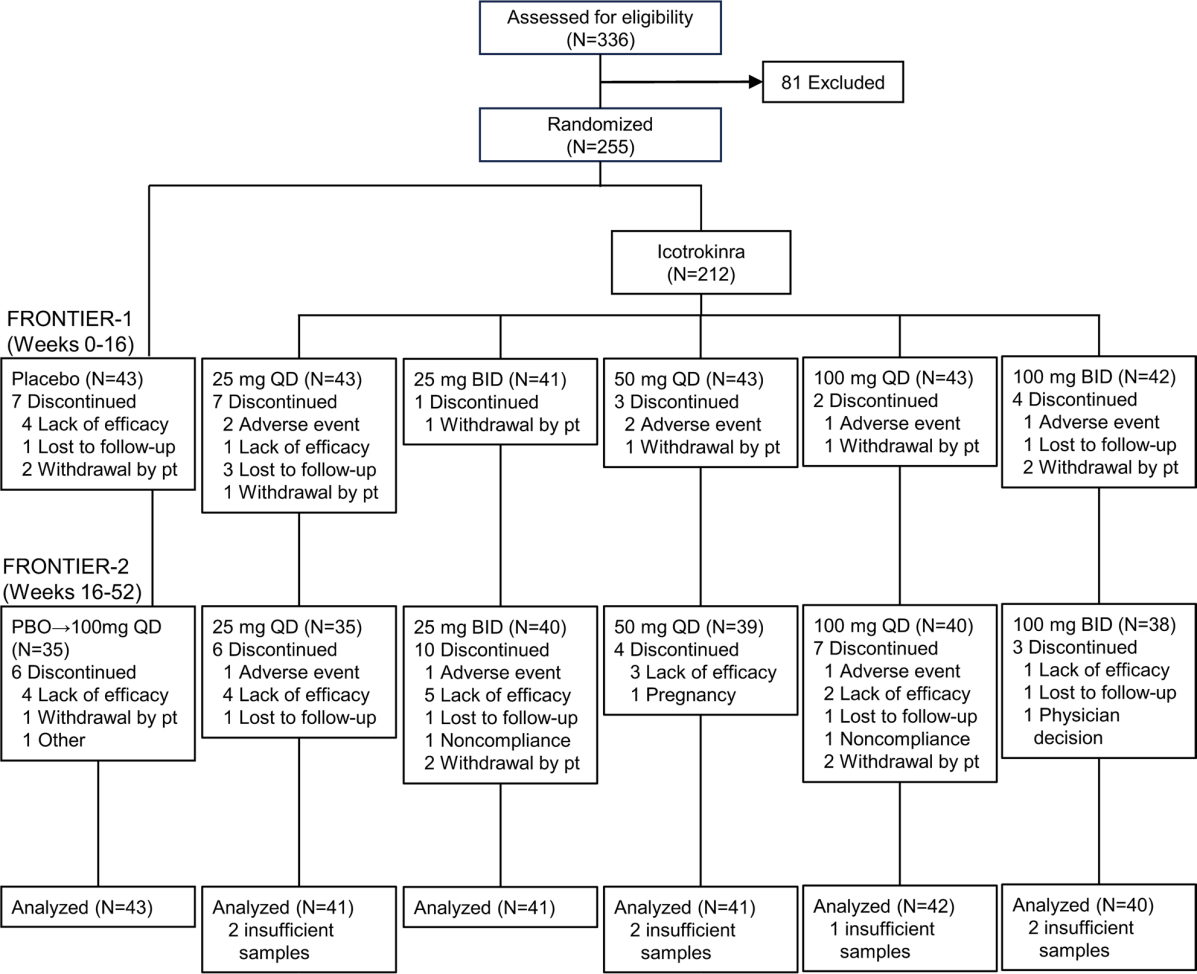
CONSORT diagram of FRONTIER-1 and FRONTIER-2 clinical trials. Flowchart illustrates the number of participants randomized to each treatment group who were included in the analyses. BID, twice daily; Pt, patient; QD, once daily.

To characterize the PD response in participants with psoriasis treated with icotrokinra, serum samples were analyzed from consenting FRONTIER-1 (*n* = 248) and FRONTIER-2 LTE (*n* = 227) participants (Figure 1). Among participants with available serum samples, no statistically significant differences were noted in baseline demographics or clinical characteristics among treatment groups, with mean age ranging from 42.0 to 45.7 years, body weight from 85.1 to 92.1 kg, and psoriasis disease duration from 15.3 to 21.7 years. Females comprised a larger proportion of participants in the placebo (41.9%) than combined icotrokinra (28.3%) groups. Also, 22.9% of participants in the combined icotrokinra dose groups had severe psoriasis, as assessed by Investigator Global Assessment (IGA), versus 11.6% in the placebo group. Serum beta-defensin-2 (BD-2) (median levels: 2,614–3,836 pg/mL), IL-17A (0.8 pg/mL for all), IL-17F (3.0–4.6 pg/mL), IL-22 (15.0–28.6 pg/mL), and IL-23 (0.5–0.6 pg/mL) levels were generally balanced across treatment groups (Table 1).

Baseline demographics were comparable, and no statistically significant differences were noted, among the 60 (29 placebo and 31 icotrokinra 100 mg twice daily [BID]) participants from whom tape-strip samples were analyzed for BD-2 (Supplemental Table 1; supplemental material available online with this article; https://doi.org/10.1172/jci.insight.193563DS1) or among the subset of 46 (20 placebo and 26 100 mg BID) FRONTIER-1 participants from whom Olink Explore HT–based broad proteomic data were generated from tape-strip samples (Table 2), with similar differences in demographics as observed in the serum cohort. Among participants with available tape-strip samples, there was a larger proportion of female participants in the placebo (55.2%) than in the 100 mg BID icotrokinra group (35.5%); 25.8% of participants in the icotrokinra dose groups had severe psoriasis, as assessed by IGA, versus 6.9% in the placebo group; however, none of these differences were statistically significant (Supplemental Table 1).

Fewer participants consented to skin biopsy sampling (placebo *N* = 3; icotrokinra *N* = 22), and as a result there were more differences in baseline characteristics of participants who provided skin biopsy samples for transcriptomic analyses (Table 3). Similar to the serum and skin-tape cohorts, there was a higher proportion of females in the placebo group (66.7%) than the icotrokinra group (18.2%). A higher proportion of icotrokinra-treated participants with skin biopsies had severe psoriasis (40.9%), while all placebo participants had moderate psoriasis, as assessed by IGA. Additionally, there were differences in prior therapies, with 36.4% of participants receiving icotrokinra receiving prior phototherapy and 0.0% in the placebo group. Findings from skin biopsy transcriptomics datasets should be interpreted within this context.

### Characterization of systemic PD changes.

**Figure 2:**
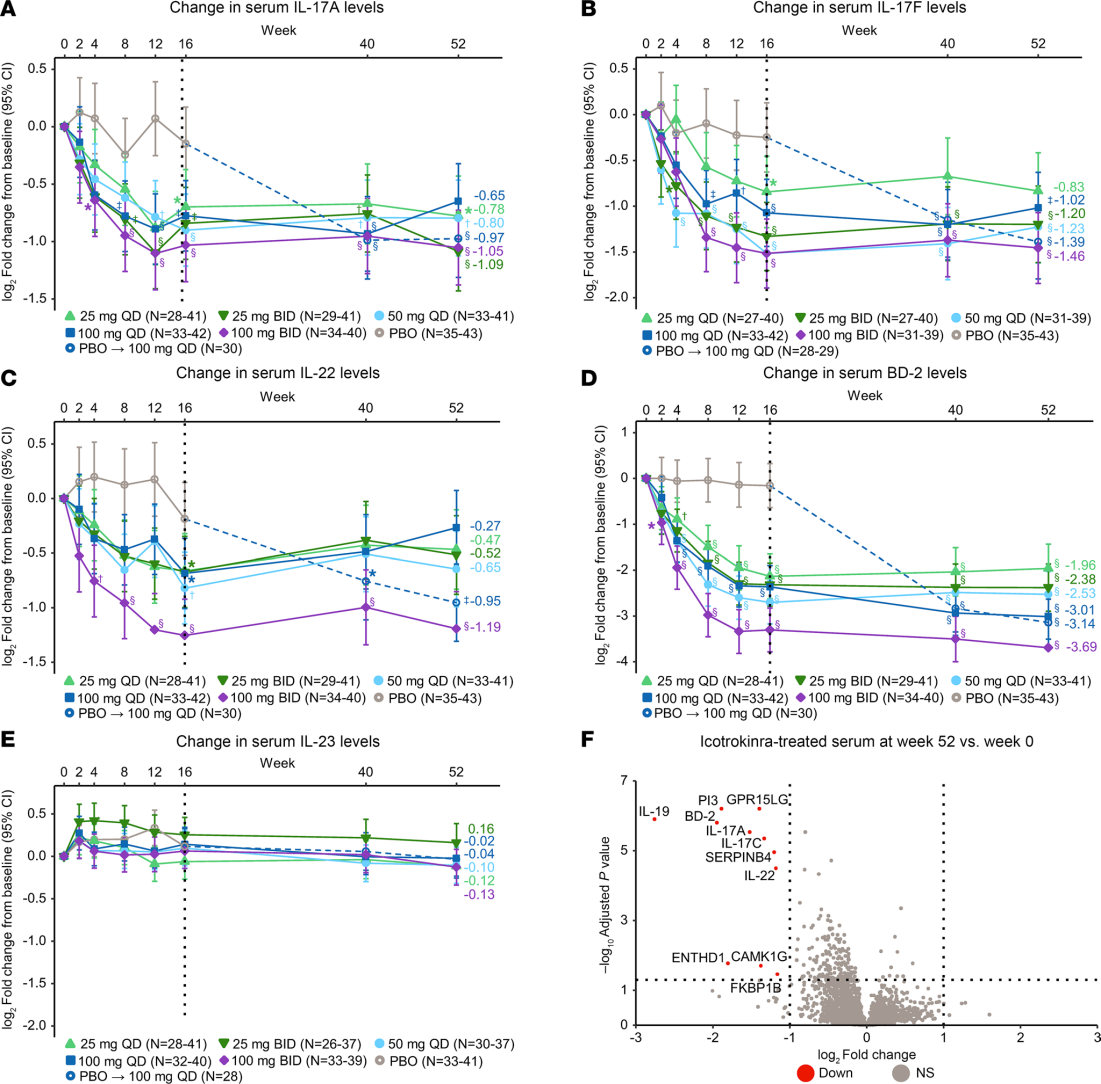
Serum levels of biomarkers relevant to psoriasis disease over time among participants receiving icotrokinra and placebo and broad protein quantification of icotrokinra-treated participant serum. Serum levels of (**A**) IL-17A, (**B**) IL-17F, (**C**) IL-22, (**D**) BD-2, and (**E**) IL-23 among participants receiving icotrokinra and placebo were compared with baseline over time. (**F**) Olink Explore HT protein quantification in serum of icotrokinra-treated participants at week 52 versus week 0. Of the 5,420 proteins that were quantified, 11 proteins were significantly downregulated (adjusted *P* < 0.05; log_2_ FC < –1) with icotrokinra after 52 weeks of treatment. (**A**–**E**) Estimated marginal means displayed. Error bars are model-based 95% confidence intervals. *Nominal *P* < 0.05 for all treatments versus baseline. ^†^Nominal *P* < 0.01 for all treatments versus baseline. ^‡^Nominal *P* < 0.001 for all treatments versus baseline. ^§^Nominal *P* < 0.0001 for all treatments versus baseline. (**F**) Log_2_ FC = 1 or –1 and adjusted *P* value = 0.05 thresholds are depicted with dotted lines. Enrichment statistics were based on Benjamini-Hochberg procedure. BD, beta-defensin; BID, twice daily; FC, fold change; PBO, placebo; PI, peptidase inhibitor; QD, once daily; CAMK1G, calcium/calmodulin dependent protein kinase IG; ENTHD1, ENTH domain-containing protein 1; FKBP1B, FKBP prolyl isomerase 1B; GPR15LG, G protein–coupled receptor 15 ligand.

Activation of the IL-23 pathway in psoriasis is associated with increased serum levels of IL-23 pathway biomarkers IL-17A, IL-17F, and IL-22 (29). Additionally, inflammatory burden in psoriatic skin has been shown to increase serum levels of antimicrobial peptides, like BD-2 (30). Serum levels of these biomarkers correlate with disease severity and treatment response, including therapies that inhibit IL-23 (30, 31). Reductions from baseline for IL-17A, IL-17F, IL-22, and BD-2 were observed for all icotrokinra doses relative to placebo, with the largest reductions in the 100 mg BID dose cohort. Importantly, reductions in serum biomarkers with icotrokinra treatment were observed as early as week 4 and were sustained through week 52, with similar reductions from week 16 to week 52 in the placebo group after transition to icotrokinra 100 mg once daily (QD; Figure 2, A–D).

Other receptor antagonist therapeutics have been shown in clinical studies to increase systemic levels of their respective ligands (32, 33). Despite reducing psoriasis-relevant biomarkers, blocking IL-23R with icotrokinra did not result in a change of serum levels of its ligand, IL-23, from baseline through week 52 (Figure 2E). Broad proteomic analysis of serum samples showed that, of the 5,420 proteins quantified on the Olink HT platform, only 11 proteins were significantly downregulated with icotrokinra after 52 weeks of treatment (FDR < 0.05; log_2_ fold change [FC] < –1; Figure 2F). Eight of these 11 downregulated proteins (PI3, IL-19, BD-2, IL-17A, IL-17C, IL-22, GPR15LG, and SERPINB4) are directly relevant to psoriasis or IL-23 pathway activation (34–38). While the log_2_ FC of the other 3 proteins (ENTHD1, CAMK1G, and FKBP1B) were less than –1, the –log_10_ *P* value was much lower than the aforementioned 8 psoriasis-relevant proteins.

Taken together these results show icotrokinra induced a dose-dependent systemic PD response with early and sustained reductions in systemic biomarkers of IL-23 pathway activation and psoriasis disease severity. Furthermore, icotrokinra selectively inhibited IL-23–driven psoriatic inflammation without broad modulation of circulating proteins, including serum IL-23 levels.

### Skin transcriptomic analysis.

**Figure 3:**
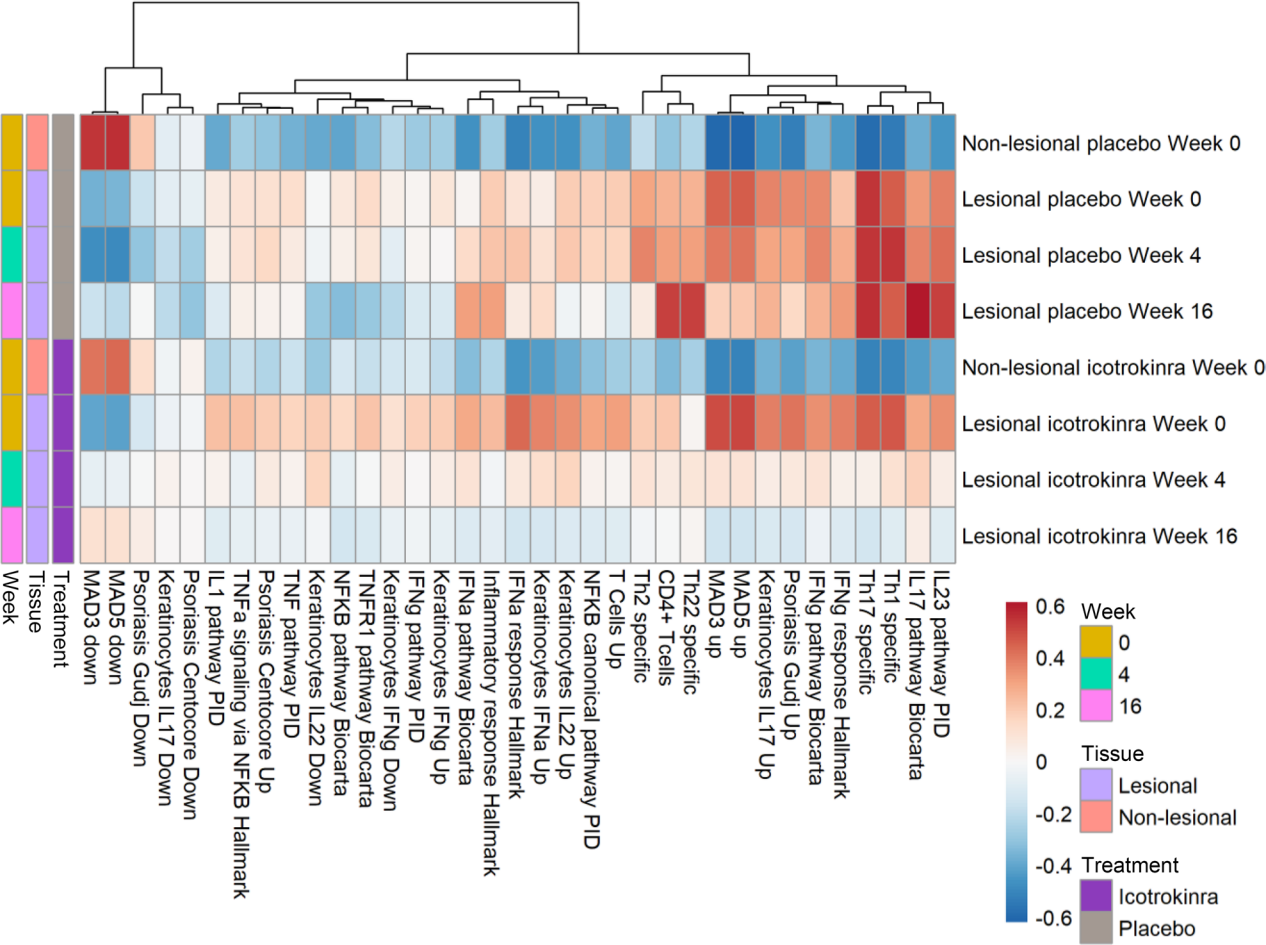
GSVA scores of skin biopsies in lesional and nonlesional skin among participants receiving icotrokinra compared with placebo. Gene set expression in lesional skin from icotrokinra-treated participants is more similar to nonlesional skin than lesional skin. GSVA, gene set variation analysis; BID, twice daily; NFKB, nuclear factor κB; PID, Pathway Interaction Database; QD, once daily; Th, T helper cells; TNF, tumor necrosis factor.

Effective treatment response in psoriasis, including IL-23 pathway inhibition, is associated with normalization of the transcriptome in lesional psoriatic skin (19). To characterize the skin PD response of icotrokinra, whole transcriptome analysis with enrichment of biological pathways and processes was performed on lesional and nonlesional skin biopsy samples. Given the limited number of participants consenting to the skin biopsy substudy, only qualitative descriptive analysis of placebo- versus pooled icotrokinra-treated participants was performed. Week 16 lesional skin samples from icotrokinra-treated, but not placebo-treated, participant samples were more similar to week 0 nonlesional skin samples (Figure 3). Icotrokinra treatment induced transcriptional changes, with modulation of gene sets associated with multiple cell types (e.g., T cells, keratinocytes) and disease-relevant cytokine signaling pathways (e.g., IL-23, IL-12, IFN-γ, and TNF-α). Importantly, icotrokinra attenuated well-characterized psoriasis-associated gene sets, including meta-analysis derived-3 (MAD-3) up and MAD-5 up, which contain genes that are upregulated in lesional compared with nonlesional psoriasis samples across multiple studies (39), as well as IL-23/IL-17 signaling. This analysis also showed increased expression of well-characterized gene sets that are downregulated in disease (e.g., MAD-3 down, MAD-5 down; Figure 3) (39). Thus, icotrokinra treatment induced transcriptional changes that are consistent with IL-23 pathway inhibition and normalization of inflammation in lesional skin.

### Characterization of skin PD changes.

Skin tape-strip sampling, a relatively minimally invasive technique, has shown promise in differentiating transcriptional signatures between psoriasis and atopic dermatitis skin (40). To further characterize icotrokinra skin PD effects, proteins derived from lesional and nonlesional skin tape-strip samples collected through week 16 were compared between participants receiving icotrokinra 100 mg BID versus placebo.

**Figure 4:**
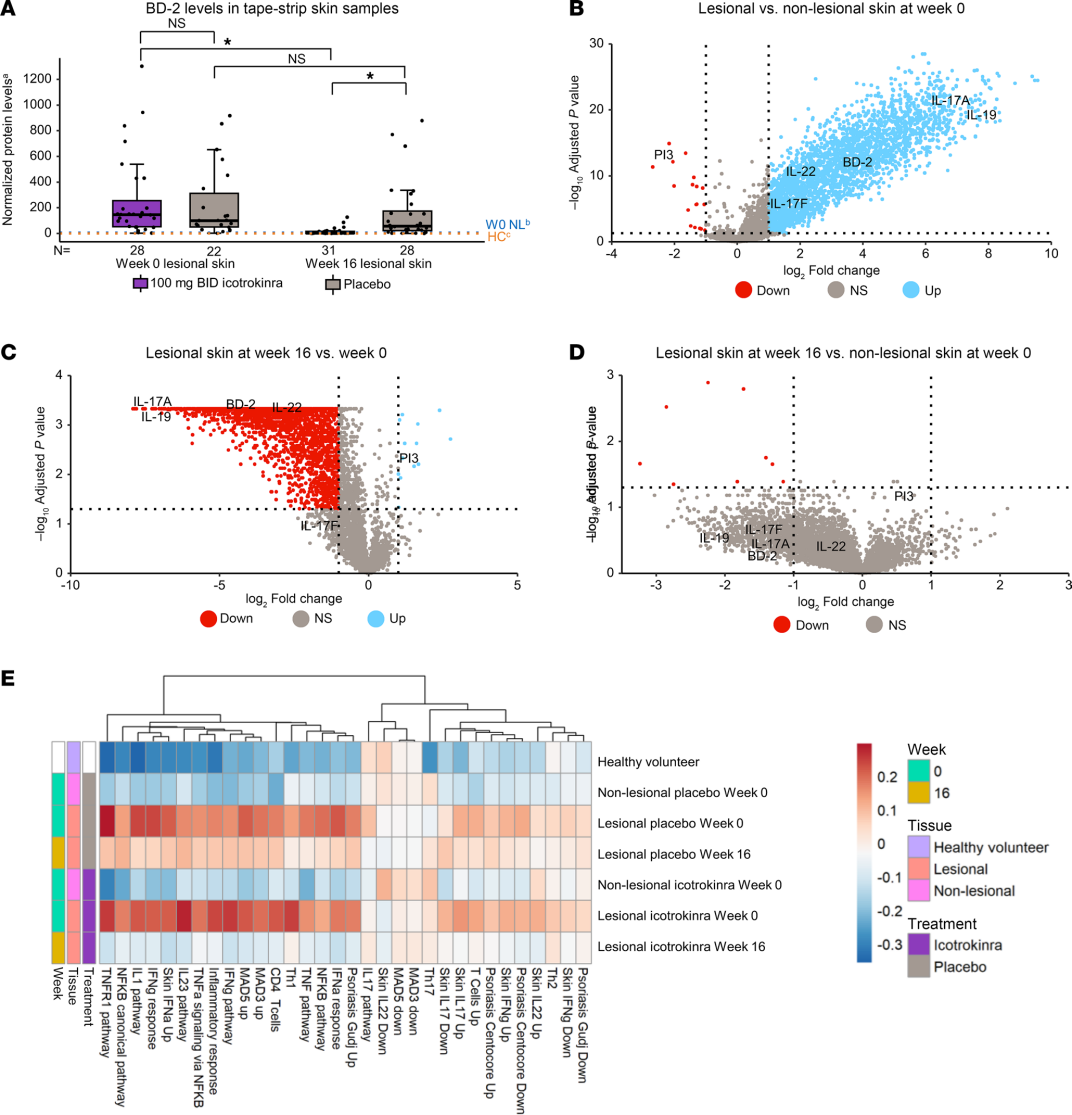
Protein changes in skin among participants receiving icotrokinra and placebo. (**A**) BD-2 protein levels in skin of participants receiving icotrokinra compared with placebo at weeks 0 and 16. Comparisons of BD-2 protein levels (pg of BD-2/μg of total protein) between lesional and nonlesional skin and between week 0 and week 16 were performed using an unpaired *t* test. BD-2 protein levels were significantly reduced after 16 weeks of icotrokinra but not with placebo. (**B**–**E**) Protein expression measured by Olink Explore HT comparing (**B**) lesional versus nonlesional skin at week 0, (**C**) lesional skin from icotrokinra-treated participants at week 16 versus week 0, and (**D**) lesional skin from icotrokinra-treated participants at week 16 versus nonlesional skin at week 0. Psoriasis-relevant proteins were upregulated at week 0 in lesional skin compared with nonlesional, and they were downregulated after 16 weeks of icotrokinra, bringing protein expression to levels comparable to nonlesional skin. (**E**) GSVA scores of protein sets in skin of placebo- and icotrokinra-treated FRONTIER-1 participants and healthy volunteers. Skin from icotrokinra-treated participants was more similar to nonlesional than lesional skin. *Nominal *P* < 0.05 based on unpaired *t* test. (**B**–**D**) Log_2_ FC = 1 or –1 and adjusted *P* value = 0.05 thresholds are depicted with dotted lines. Enrichment statistics were based on Benjamini-Hochberg procedure. ^a^BD-2 pg/mL: total μg/mL. ^b^Average of 100 mg BID and placebo (PBO) nonlesional skin at week 0. ^c^Healthy control (HC) skin tape-strip samples were obtained from sponsor healthy donor program at Johnson & Johnson (Protocol NOCOMPOUNDNAP1001). DEFB4, defensin beta 4; BID, twice daily; NFKB, nuclear factor κB; NL, nonlesional; PI, peptidase inhibitor; RQ, relative quantification; Th, T helper cells; TNF, tumor necrosis factor; W, week.

The sensitivity of tape-strip based analysis was assessed by evaluating changes in skin levels of BD-2, a serum biomarker responsive to icotrokinra (Figure 2D). BD-2 protein levels were elevated in week 0 lesional skin in both icotrokinra 100 mg BID and placebo groups. Icotrokinra 100 mg BID treatment, but not placebo, significantly reduced BD-2 levels in lesional skin to levels approaching those seen in nonlesional and healthy control skin (Figure 4A).

To further characterize changes in proteins collected via tape-strip sampling, broad proteomics was performed using the Olink Explore HT platform (>5,420 proteins assessed). At baseline, the protein landscape in lesional tape-strip samples was markedly differentiated from nonlesional samples, with enrichment of several psoriasis-relevant proteins. A total of 2,684 proteins were upregulated and 18 proteins were downregulated in lesional samples relative to nonlesional samples. The upregulated proteins included known IL-23/IL-17 pathway biomarkers (IL-19, IL-17A, BD-2, IL-22, and IL-17F; Figure 4B). Similar differences in protein expression patterns were also observed when comparing week 0 lesional skin with skin samples from healthy volunteers (Supplemental Figure 1). A paired analysis of week 16 versus week 0 lesional samples showed that 100 mg BID icotrokinra treatment downregulated disease-relevant proteins, with 2,366 downregulated and 13 upregulated proteins after 16 weeks of treatment (Figure 4C). Importantly, a paired analysis of week 16 lesional versus week 0 nonlesional samples in icotrokinra-treated participants showed 5,406 out of 5,415 proteins were comparable at week 16, and of the 9 proteins that were different between these groups, none are known to be related to the pathogenesis of psoriasis (Figure 4D).

To further evaluate the impact of icotrokinra on psoriasis-relevant proteins, protein sets were generated from well-characterized or published psoriasis and IL-23 pathway activation gene sets (39, 41–43). In this GSVA, the icotrokinra-treated week 16 lesional samples were more similar to healthy control and nonlesional samples than both week 0 lesional and placebo-treated week 16 lesional samples (Figure 4E). Thus, icotrokinra normalized psoriasis-associated protein signatures in lesional skin to levels observed in nonlesional skin by week 16, suggesting normalization of skin inflammation with treatment.

### Correlation of icotrokinra PD response with observed clinical response and exposure.

**Figure 5:**
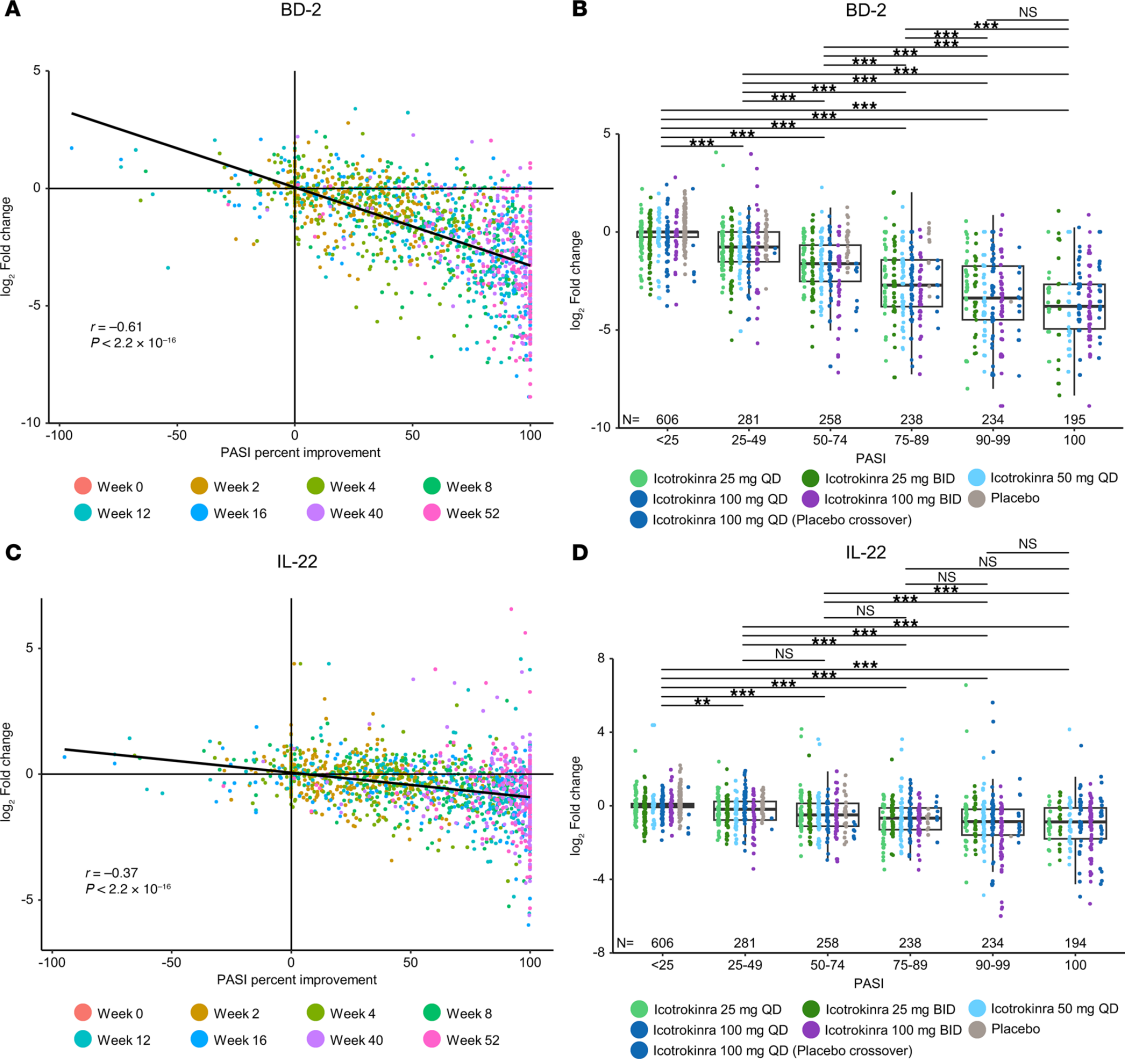
Levels of BD-2 and IL-22 in serum are correlated with PASI response among participants receiving icotrokinra. (**A** and **B**) BD-2. (**C** and **D**) IL-22.Correlation between PASI change from baseline and biomarker change from baseline was performed using Pearson’s correlation. BD-2 and IL-22 log_2_ FC from baseline were compared across all PASI percentage improvement categories, and adjusted *P* values were generated using Tukey’s multiple comparisons of means. Reductions in BD-2 and IL-22 were correlated with PASI improvement. **P < 0.01. ***P < 0.001. Correlation performed using Pearson’s correlation. Adjusted *P* values calculated using Tukey’s test for multiple comparisons of means. BD, beta-defensin; BID, twice daily; PASI, Psoriasis Area and Severity Index; QD, once daily.

To understand the exposure-response relationship of icotrokinra PD and its correlation with clinical response, the pharmacokinetic (PK)/PD relationship coupled with clinical response was assessed. Log_2_ FC in serum BD-2 levels strongly correlated with percentage PASI improvement (*R* = –0.6, *P* < 2.2 × 10^–16^; Figure 5A). Furthermore, higher reductions in serum BD-2 levels were associated with higher levels of PASI improvement (Figure 5B). Similar results were obtained with IL-22, with serum levels correlating with PASI response (*R* = –0.4, *P* < 2.2 × 10^–16^; Figure 5, C and D).

**Figure 6:**
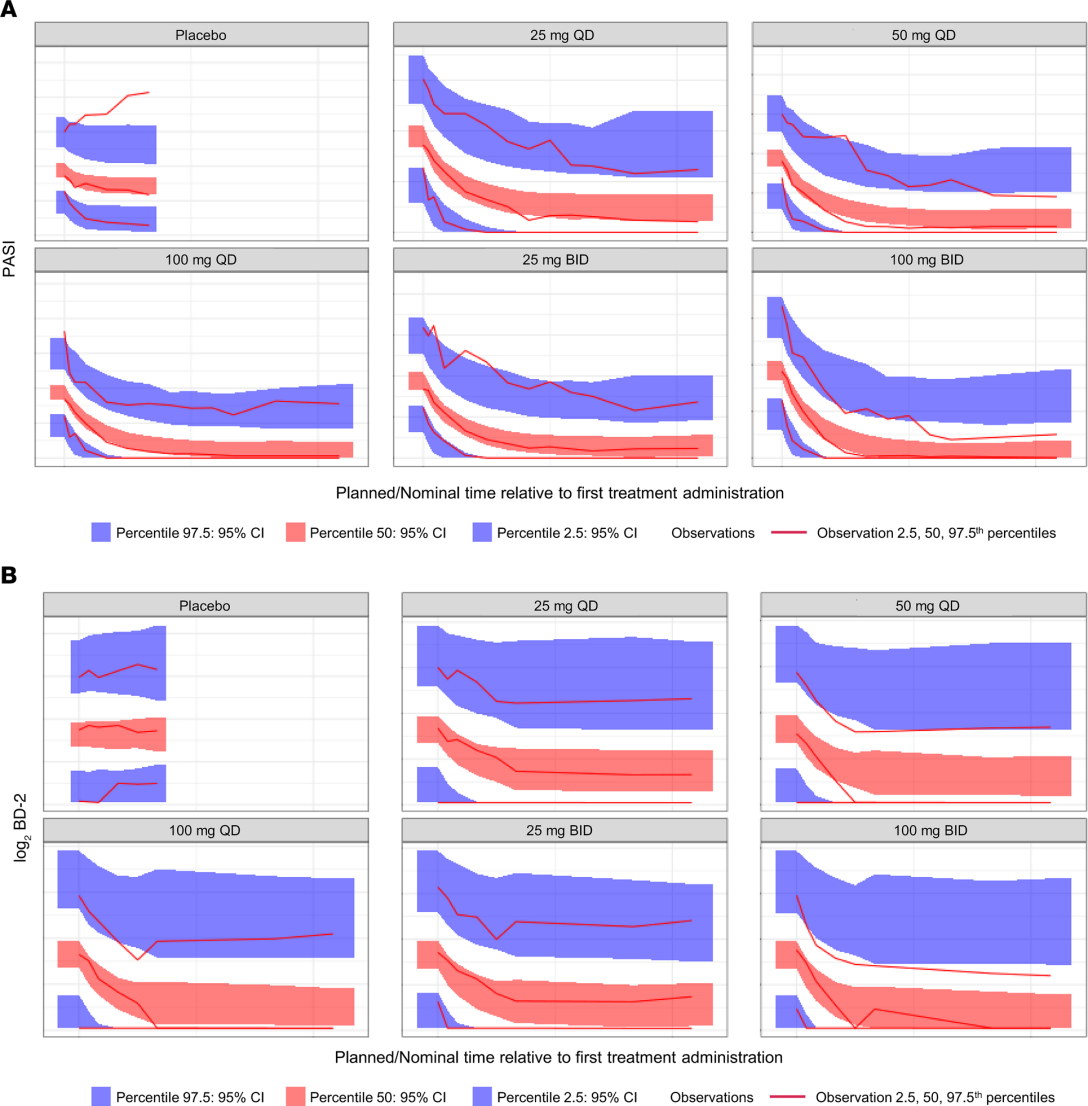
Visual predictive check for longitudinal population PK/PD model describing the effect of icotrokinra on PASI and log_2_ BD-2. (**A**) PASI. (**B**) log_2_ BD-2. BD, beta-defensin; BID, twice daily; CI, confidence interval; PASI, Psoriasis Area and Severity Index; PD, pharmacodynamics; PK, pharmacokinetics; QD, once daily.

Population PK/PD modeling was performed to evaluate the relationship between icotrokinra treatment on PASI scores and serum BD-2 levels over time. Sequential population PK/PD modeling with indirect response models for PASI scores and log_2_ BD-2 levels fit the data well (visual predictive check based on simulation of *N* = 1,000 shown in Figure 6). The precision of the parameter estimate for the concentration at half of the maximum effect (EC_50_) improved slightly by including BD-2 levels in the model, compared with a model including PASI scores alone. The time course and magnitude of effect of icotrokinra concentrations on PASI scores and BD-2 levels were similar. These results demonstrate that the exposure-clinical response and exposure-biomarker relationships were similar with icotrokinra. Thus, there was a strong correlation between exposure, PD changes, and the observed clinical response with icotrokinra.

## Discussion

Use of conventional oral therapies approved for psoriasis can be constrained by lower efficacy and safety/tolerability concerns (7). Monoclonal antibodies targeting the IL-23 p19 subunit or the IL-12/23 p40 subunit have been shown to be safe and highly efficacious in treating psoriasis (20, 22, 44, 45); however, many patients prefer oral therapies or have needle phobia, which limit the overall use of injectable biologics (46). The more recently approved advanced oral therapies for psoriasis, apremilast and deucravacitinib, have demonstrated lower efficacy or safety/tolerability concerns relative to biologics (9, 10). While several psoriasis treatments block the IL-23 pathway by targeting the IL-23 cytokine ligand, currently no available drugs bind the IL-23R. In the FRONTIER-1 and -2 studies, icotrokinra, the first and only targeted oral peptide that selectively binds IL-23R, demonstrated high rates of durable skin clearance through 52 weeks in participants with moderate-to-severe plaque psoriasis. In these studies, rates of adverse events were similar across the icotrokinra and placebo groups, and no additional adverse events or safety signals were identified through 52 weeks of treatment (27, 28). Preclinical studies also found no risk of drug-drug interactions and no major metabolites (26). The present study showed that selective targeting of IL-23R signaling with icotrokinra induced an early and sustained PD response in participants with psoriasis.

Since IL-23R has not been previously targeted for therapeutic efficacy, it was important to confirm that icotrokinra induced PD changes, both in the systemic circulation and the target skin tissue in patients with psoriasis. Oral dosing of icotrokinra, as early as week 4, substantially reduced psoriasis-relevant biomarkers in serum across all dosing regimens compared with placebo. In line with clinical responses seen in FRONTIER-1 and -2 studies, the greatest reductions in serum biomarkers were observed with the icotrokinra 100 mg BID regimen. This systemic PD response with icotrokinra was dose dependent and sustained through week 52. While some receptor antagonists have been shown to increase their respective ligand levels in serum (32, 33), icotrokinra induced these systemic PD changes without increasing serum levels of IL-23 through 1 year of treatment. Previous studies showed that icotrokinra potently and selectively inhibits IL-23 signaling (25), and the results presented here further confirm that icotrokinra selectively blocks IL-23–driven inflammation in patients with psoriasis without broader impacts on circulating proteins.

In addition to these systemic PD changes, icotrokinra drove PD changes in the target tissue with attenuation of transcriptional skin biomarkers that are relevant to disease and IL-23 pathway activation in psoriasis. Furthermore, icotrokinra downregulated psoriasis-associated proteins in tape strips of lesional skin to levels observed in nonlesional skin. Icotrokinra’s PD response was positively correlated with increasing exposures, as well as the clinical response, suggesting a strong relationship between the PD and clinical exposure-response. Ongoing phase III trials of icotrokinra (ICONIC-LEAD [NCT06095115], ICONIC-TOTAL [NCT06095102], ICONIC-ADVANCE 1 [NCT06143878], and ICONIC-ADVANCE 2 [NCT06220604]) in broad populations of adults with moderate-to-severe plaque psoriasis will further confirm and provide additional details regarding safety and efficacy profiles, including patient reported outcomes and how they correlate to biomarker changes in larger patient cohorts.

In addition to defining the magnitude of skin PD response with icotrokinra, these analyses suggests that tape-strip sampling can provide a minimally invasive alternative to skin biopsies for evaluating disease biology and treatment response in participants with psoriasis. Tape-strip sampling offers several advantages over full-thickness biopsies, including its minimally invasive technique and ease of sample collection, making it a more convenient option for patients. Additionally, tape-stripping allows for real-time analysis of changes in the skin, making it suitable for monitoring disease progression or treatment responses.

The results reported here have some limitations. In addition to reported numerical differences in baseline characteristics of participants in this serum and tissue biomarker analysis, only a small number of participants consented to the optional skin biopsy substudy. As such, there were more differences in baseline characteristics of participants who consented to skin biopsy sampling, and results should be interpreted within this context. Due to these limitations, reported skin transcriptional changes were qualitative; therefore, no quantitative interpretations among dosing regimens or the magnitude of the effect could be drawn. Tape-strip sampling also has some notable limitations, including that it only samples the outer epidermal layers, so deeper layers of skin are not profiled as can be done with full-thickness biopsies. Additionally, similar to skin biopsy collections, tape-strip sampling could be influenced by factors such as operator technique and skin condition. Despite these limitations, our results clearly show that icotrokinra selectively induced dose-dependent systemic PD responses, characterized by early (week 4) and sustained (week 52) reductions in objective biomarkers of IL-23 pathway activation and disease severity. Icotrokinra also attenuated psoriasis biomarkers to normalize inflammation in the skin and drive disease improvement in participants with psoriasis. Taken together, the data strongly suggest that icotrokinra has a PD profile that is consistent with its mechanism of action of selectively inhibiting IL-23–driven inflammation and has the potential to be an oral therapy that will broaden the treatment options for patients with psoriasis.

## Methods

### Sex as a biological variable

Sex was not considered as a biological variable.

### Patients and trial design

FRONTIER-1 study design, patient eligibility criteria, and initial results have been previously reported (27). Briefly, FRONTIER-1 participants with moderate-to-severe plaque psoriasis were randomized 1:1:1:1:1:1 to receive icotrokinra at a dose of 25 mg QD, 25 mg BID, 50 mg QD, 100 mg QD, 100 mg BID, or placebo for 16 weeks. FRONTIER-2 was the LTE study, during which participants randomized to icotrokinra continued their assigned treatment through week 52 and those receiving placebo crossed over to 100 mg QD icotrokinra after week 16 (47). Concomitant treatments for psoriasis were prohibited, except for shampoos containing salicylic acid and nonmedicated topical moisturizers.

### Serum biomarker analyses

Blood samples were collected through week 52. Serum IL-17A, IL-17F, and IL-23 concentrations were measured using commercially available kits from EMD Millipore Corporation and analyzed on the SMCxPRO instrument. The IL-22 assay was developed internally on the SMC platform and run on the SMCxPRO instrument. A custom BD-2 assay, developed by Mesoscale Discovery (MSD), was performed according to the manufacturer’s protocol and read on the MESO QuickPlex SQ120 MSD instrument. A broad evaluation of protein expression was also conducted on these serum samples, using the Olink Explore HT platform (Olink Proteomics).

### Skin tape-strip proteomic analysis

The protocol for protein extraction from tape-strip samples was optimized using the method described by Clausen et al. (48). See Supplemental Methods for protocol details. Briefly, 20 tape-strips were collected from lesional or nonlesional skin using D-squame tape discs at each sampling time point (lesional and nonlesional at baseline; lesional at week 16). Protein was extracted from the last 4 tape-strips of each patient to sample the deepest layers of skin. BD-2 and broad proteomics were performed using MSD and Olink Explore HT platform assays per manufacturer’s protocol.

### Skin biopsy processing for bulk RNA sequencing

Skin biopsies for RNA sequencing were collected from a subset of patients. See Supplemental Methods for protocol details.

### Statistics

#### Participant and disease characteristic differences in baseline characteristics.

Participant and disease characteristics at baseline were compared between placebo and combined icotrokinra participants with available serum samples using 1-way ANOVA for continuous variables and χ^2^ for categorical variables. Due to small sample sizes, comparisons between placebo and 100 mg BID icotrokinra participants with available Olink tape-strip data were performed using Kruskal-Wallis for continuous variables and χ^2^ for categorical variables, except baseline IGA 3/4, whose *P* value was calculated using Fisher’s exact test.

#### PD treatment effect on biomarker levels in serum.

Log_2_ FC from baseline and comparisons between all icotrokinra doses and baseline for serum biomarkers through week 52 were calculated directly using a linear mixed effects model with main and interaction fixed effects of treatment arm, time, and participant random effect, using post hoc estimated marginal mean.

#### Olink proteomic analysis.

Differential protein analyte analysis was performed using the R package OlinkAnalyze (DOI: 10.32614/CRAN.package.OlinkAnalyze). Statistical testing was performed using the olink_ttest function from this package, which applied a Welch 2-sample *t* test or paired *t* test at confidence level 0.95 for every protein for a given grouping variable, correcting for multiple testing using the Benjamini-Hochberg method. Volcano plots for both serum and tape-strip samples were generated in R using ggplot2 with intensity-normalized protein expression values. GSVA was also performed on Olink proteomic data from tape-strip samples by generating protein sets from the gene sets described under the transcriptomic analysis.

#### Skin transcriptomic analysis.

RNA sequencing data analysis and GSVA were performed with the GSVA R package (49). Gene sets used for GSVA were from published data (39, 41–43), BioCarta pathways, and MSigDB hallmark pathways. Given the limited number of individuals participating in the skin biopsy substudy, only pooled icotrokinra versus placebo analysis was performed.

#### Correlation of biomarker levels and disease activity.

Data from weeks 0–52 for all participants were pooled, and PASI percentage improvement was evaluated for correlation with BD-2 and IL-22 serum levels using Pearson’s correlation. These data were also grouped into 6 PASI percentage improvement categories, <25%, 25%–49%, 50%–74%, 75%–89%, 90%–99%, and 100%, and adjusted P values were calculated using Tukey’s test for multiple comparisons of means.

#### Population PK and PD modeling.

Data from all FRONTIER-1 and -2 participants were included in this analysis. Indirect response models involving a simultaneous fit of BD-2 levels and PASI scores were used to describe the longitudinal population PK/PD of the PASI and biomarker time courses having a common drug concentration resulting in EC_50_. The model for the drug effect on continuous PASI accounted for a placebo effect.

### Study approval

The study was approved by each site’s institutional review board(s)/ethics committee for all sites. Written informed consent was obtained from all participants prior to their participation in the study.

### Data availability

The data sharing policy of Johnson & Johnson is available at http://www.janssen.com/clinical-trials/transparency As noted on this site, requests for access to the study data can be submitted through the Yale Open Data Access (YODA) Project site at http://yoda.yale.edu The data supporting the findings of this study may be obtained from the authors upon reasonable request.

Data supporting the figures can be found in the Supporting Data Values file.

## Author contributions

Study design was done by JGK, RB, KE, LKF, ASP, AP, CD, PN, DMW, MM, TO, YWY, MWLL, LSM, CAC, BM, D Ruane, and AKK. Performing experiments was done by DS, KP, DH, SB, LPT, CHC, RP, and JJR. Acquiring data was done by DS, KP, DH, SB, LPT, CHC, and RP. Analyzing data was done by DS, KP, DH, SB, LPT, CHC, RP, D Richards, EYC, MEP, JS, EB, YX, WZ, and BEK. Interpreting data was done by JGK, RB, KE, LKF, ASP, AP, CD, PN, DMW, MM, TO, YWY, MWLL, LSM, CAC, BM, D Ruane, DS, KP, DH, SB, LPT, CHC, RP, D Richards, EYC, MEP, JS, EB, YX, WZ, BEK, AKK, and JJR. Writing, reviewing, and revising the manuscript were done by JGK, RB, KE, LKF, ASP, AP, JJR, CD, PN, DMW, MM, TO, YWY, MWLL, LSM, CAC, BM, D Ruane, and AKK.

## Funding support

- Johnson & Johnson.

## Supplementary Material

Supplemental data

ICMJE disclosure forms

Supporting data values

## Figures and Tables

**Table 1:**
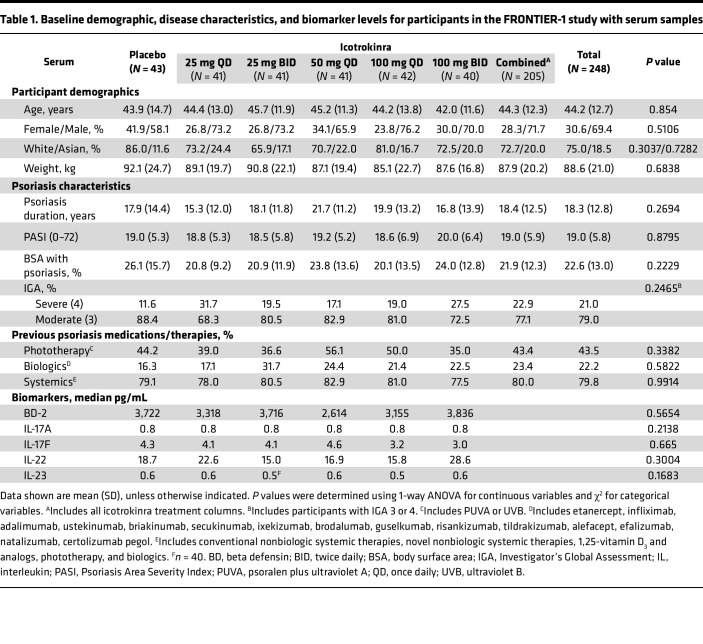
Baseline demographic, disease characteristics, and biomarker levels for participants in the FRONTIER-1 study with serum samples

**Table 2:**
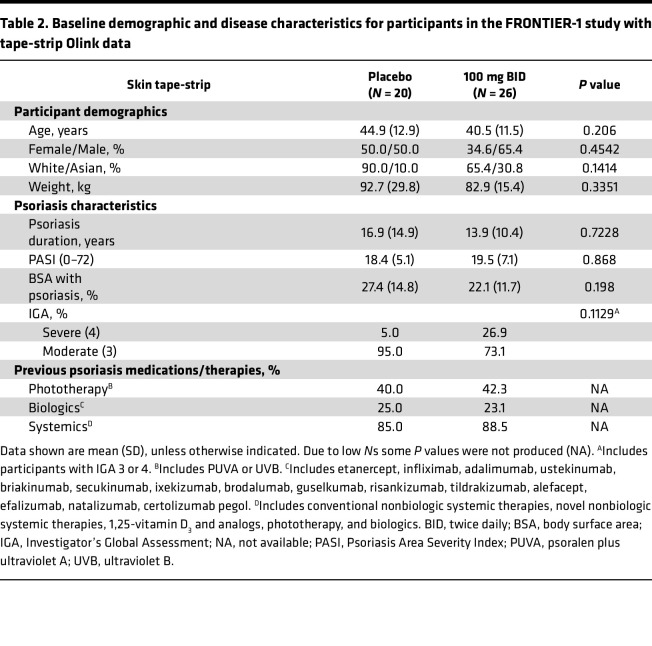
Baseline demographic and disease characteristics for participants in the FRONTIER-1 study with tape-strip Olink data

**Table 3:**
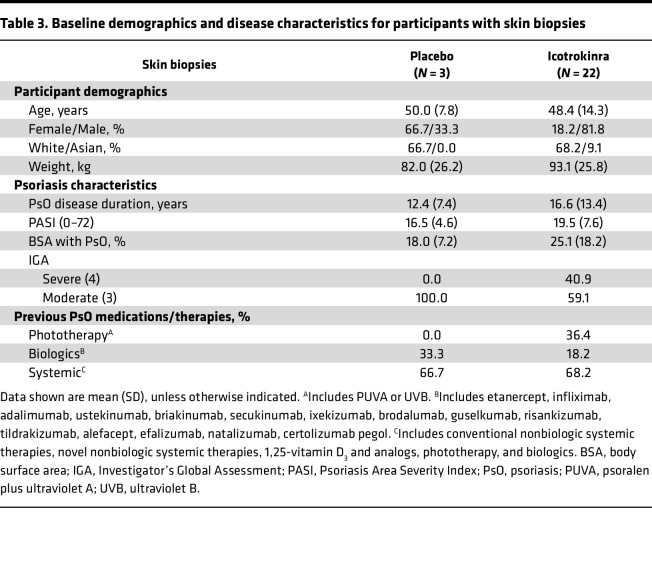
Baseline demographics and disease characteristics for participants with skin biopsies

## Version 1 12/22/2025

Electronic publication

## References

[1] Parisi R (2013). Global epidemiology of psoriasis: a systematic review of incidence and prevalence. J Invest Dermatol.

[2] Boehncke WH, Schön MP (2015). Psoriasis. Lancet.

[3] Kim J, Krueger JG (2017). Highly effective new treatments for psoriasis target the IL-23/type 17 T cell autoimmune axis. Annu Rev Med.

[4] Goldenberg G (2016). New oral therapies for psoriasis: a comprehensive review. J Clin Aesthet Dermatol.

[5] Lebwohl M (2022). Evolution of patient perceptions of psoriatic disease: results from the understanding psoriatic disease leveraging insights for treatment (UPLIFT) survey. Dermatol Ther (Heidelb).

[6] Menter A (2019). Joint AAD-NPF guidelines of care for the management and treatment of psoriasis with biologics. J Am Acad Dermatol.

[7] Sbidian E (2022). Systemic pharmacological treatments for chronic plaque psoriasis: a network meta-analysis. Cochrane Database Syst Rev.

[8] Armstrong AW (2020). Comparison of biologics and oral treatments for plaque psoriasis: a meta-analysis. JAMA Dermatol.

[9] Strober B (2023). Deucravacitinib versus placebo and apremilast in moderate to severe plaque psoriasis: efficacy and safety results from the 52-week, randomized, double-blinded, phase 3 program for evaluation of TYK2 inhibitor psoriasis second trial. J Am Acad Dermatol.

[10] Armstrong AW (2023). Short-, mid-, and long-term efficacy of deucravacitinib versus biologics and nonbiologics for plaque psoriasis: a network meta-analysis. Dermatol Ther (Heidelb).

[11] Cargill M (2007). A large-scale genetic association study confirms IL12B and leads to the identification of IL23R as psoriasis-risk genes. Am J Hum Genet.

[12] Liu Y (2008). A genome-wide association study of psoriasis and psoriatic arthritis identifies new disease loci. PLoS Genet.

[13] Constantinides MG (2019). MAIT cells are imprinted by the microbiota in early life and promote tissue repair. Science.

[14] Gaffen SL (2014). The IL-23-IL-17 immune axis: from mechanisms to therapeutic testing. Nat Rev Immunol.

[15] Mehta H (2021). Differential changes in inflammatory mononuclear phagocyte and T-cell profiles within psoriatic skin during treatment with guselkumab vs. secukinumab. J Invest Dermatol.

[16] Krueger JG (2024). IL-23 past, present, and future: a roadmap to advancing IL-23 science and therapy. Front Immunol.

[17] Polese B (2020). Innate lymphocytes in psoriasis. Front Immunol.

[18] Pawlak M (2022). Induction of a colitogenic phenotype in Th1-like cells depends on interleukin-23 receptor signaling. Immunity.

[19] Blauvelt A (2024). Residual lesional gene expression in psoriasis patients with complete skin clearance treated with guselkumab or adalimumab in VOYAGE 1 and 2. J Invest Dermatol.

[20] Langley RG (2018). Efficacy and safety of guselkumab in patients with psoriasis who have an inadequate response to ustekinumab: results of the randomized, double-blind, phase III NAVIGATE trial. Br J Dermatol.

[21] McInnes IB (2022). Long-term efficacy and safety of guselkumab, a monoclonal antibody specific to the p19 subunit of interleukin-23, through two years: results from a phase III, randomized, double-blind, placebo-controlled study conducted in biologic-naive patients with active psoriatic arthritis. Arthritis Rheumatol.

[22] Papp KA (2008). Efficacy and safety of ustekinumab, a human interleukin-12/23 monoclonal antibody, in patients with psoriasis: 52-week results from a randomised, double-blind, placebo-controlled trial (PHOENIX 2). Lancet.

[23] Reich K (2017). Tildrakizumab versus placebo or etanercept for chronic plaque psoriasis (reSURFACE 1 and reSURFACE 2): results from two randomised controlled, phase 3 trials. Lancet.

[24] Reich K (2021). Five-year maintenance of clinical response and health-related quality of life improvements in patients with moderate-to-severe psoriasis treated with guselkumab: results from VOYAGE 1 and VOYAGE 2. Br J Dermatol.

[25] Fourie AM (2024). JNJ-77242113, a highly potent, selective peptide targeting the IL-23 receptor, provides robust IL-23 pathway inhibition upon oral dosing in rats and humans. Sci Rep.

[26] Knight B (2025). Translational pharmacokinetics of icotrokinra, a targeted oral peptide that selectively blocks interleukin-23 receptor and inhibits signaling. Dermatol Ther (Heidelb).

[27] Bissonnette R (2024). An oral interleukin-23-receptor antagonist peptide for plaque psoriasis. N Engl J Med.

[28] Ferris LK (2025). FRONTIER-2: a phase 2b, long-term extension, dose-ranging study of oral JNJ-77242113 for the treatment of moderate-to-severe plaque psoriasis. J Am Acad Dermatol.

[29] Siebert S (2024). Modulation of interleukin-23 signaling with guselkumab in biologic-naive patients versus tumor necrosis factor inhibitor-inadequate responders with active psoriatic arthritis. Arthritis Rheumatol.

[30] Kolbinger F (2017). β-defensin 2 is a responsive biomarker of IL-17A-driven skin pathology in patients with psoriasis. J Allergy Clin Immunol.

[31] Blauvelt A (2024). Differential pharmacodynamic effects on psoriatic biomarkers by guselkumab versus secukinumab correlate with long-term efficacy: an ECLIPSE substudy. JID Innov.

[32] De Soyza A (2015). A randomised, placebo-controlled study of the CXCR2 antagonist AZD5069 in bronchiectasis. Eur Respir J.

[33] Mitroi GG (2024). Exploring the potential of IL-4 and IL-13 plasma levels as biomarkers in atopic dermatitis. Life (Basel).

[34] Chan TC (2018). Interleukin 23 in the skin: role in psoriasis pathogenesis and selective interleukin 23 blockade as treatment. Ther Adv Chronic Dis.

[35] Deng J (2023). Multi-omics approach identifies PI3 as a biomarker for disease severity and hyper-keratinization in psoriasis. J Dermatol Sci.

[36] Chen C (2024). GPR15LG regulates psoriasis-like inflammation by down-regulating inflammatory factors on keratinocytes. Biosci Rep.

[37] Takeda A (2002). Overexpression of serpin squamous cell carcinoma antigens in psoriatic skin. J Invest Dermatol.

[38] Zhang Y (2023). SERPINB4 promotes keratinocyte inflammation via p38MAPK signaling pathway. J Immunol Res.

[39] Tian S (2012). Meta-analysis derived (MAD) transcriptome of psoriasis defines the “core” pathogenesis of disease. PLoS One.

[40] He H (2021). Tape strips detect distinct immune and barrier profiles in atopic dermatitis and psoriasis. J Allergy Clin Immunol.

[41] Mee JB (2007). The psoriatic transcriptome closely resembles that induced by interleukin-1 in cultured keratinocytes: dominance of innate immune responses in psoriasis. Am J Pathol.

[42] Nograles KE (2008). Th17 cytokines interleukin (IL)-17 and IL-22 modulate distinct inflammatory and keratinocyte-response pathways. Br J Dermatol.

[43] Swindell WR (2013). Dissecting the psoriasis transcriptome: inflammatory- and cytokine-driven gene expression in lesions from 163 patients. BMC Genomics.

[44] Blauvelt A (2017). Efficacy and safety of guselkumab, an anti-interleukin-23 monoclonal antibody, compared with adalimumab for the continuous treatment of patients with moderate to severe psoriasis: results from the phase III, double-blinded, placebo- and active comparator-controlled VOYAGE 1 trial. J Am Acad Dermatol.

[45] Reich K (2017). Efficacy and safety of guselkumab, an anti-interleukin-23 monoclonal antibody, compared with adalimumab for the treatment of patients with moderate to severe psoriasis with randomized withdrawal and retreatment: results from the phase III, double-blind, placebo- and active comparator-controlled VOYAGE 2 trial. J Am Acad Dermatol.

[46] Duncanson E (2021). The prevalence and evidence-based management of needle fear in adults with chronic disease: a scoping review. PLoS One.

[47] Ferris LK (2025). FRONTIER-2: A phase 2b, long-term extension, dose-ranging study of oral JNJ-77242113 for the treatment of moderate-to-severe plaque psoriasis. J Am Acad Dermatol.

[48] Clausen ML (2016). Tape stripping technique for stratum corneum protein analysis. Sci Rep.

[49] Hanzelmann S (2013). GSVA: gene set variation analysis for microarray and RNA-seq data. BMC Bioinformatics.

