# Supplementary material for: Icotrokinra induces early and sustained pharmacodynamic responses in phase IIb study of patients with moderate-to-severe psoriasis
Source: JCI Insight. 2025 Dec 22;10(24):e193563. doi: 10.1172/jci.insight.193563 (PMC12890502; doi:10.1172/jci.insight.193563)
Supplement: Supplemental data [file jciinsight-10-193563-s010.pdf]

Supplemental material

Supplemental methods

Participant Population

FRONTIER-1 study design, patient eligibility criteria, and initial results have been previously reported (27). Briefly, FRONTIER-1 participants with moderate-to-severe plaque psoriasis were randomized 1:1:1:1:1:1 to receive icotrokinra at a dose of 25 mg once daily (QD), 25 mg twice daily (BID), 50 mg QD, 100 mg QD, 100 mg BID, or placebo for 16 weeks. FRONTIER-2 was the long-term extension (LTE) study, during which participants randomized to icotrokinra continued their assigned treatment through Week 52 and those receiving placebo crossed over to 100 mg QD icotrokinra after Week 16.

Serum biomarker analyses

Blood samples were collected through Week 52. Serum protein concentrations were measured using commercially available (EMD Millipore Corporation) analytes for IL-17A, IL-17F, and IL-23. Manufacturer protocols were followed, and readings were obtained using the SMCxPRO® immunoassay instrument platform. The IL-22 assay was developed internally as a coated plate-based assay, based on the SMC™ platform and run on the SMCxPRO assay instrument. A custom BD-2 assay, which was developed by MSD using their patented electrochemiluminescence-based technology, was performed according to the manufacturer's protocol; assay plates were read on the MESO QuickPlex SQ120 MSD instrument. For all assays, adjustments were made to protein concentrations measuring below the empirically defined lower limit of quantification (LLOQ) and above the upper limit of quantification

(ULOQ). Concentration values below the LLOQ were adjusted to 0.5 x LLOQ, and those exceeding the ULOQ were adjusted to 1.25 x ULOQ, as reported previously (30,51). Additionally, all sample concentration values were multiplied by their respective sample dilution factors. A broad evaluation of protein expression was also conducted on these serum samples, using the Olink® Explore HT platform (Olink Proteomics).

Skin tape-strip proteomic analysis

Protein was extracted from 4 tape strips per participant using D-squame tape discs at each sampling time point (lesional and non-lesional at baseline; lesional at Week 16) with chilled lysis buffer (0.05% Tween-20 DPBS with protease inhibitors) using an ultrasonicator (35kHz for 15 minutes). Total protein concentrations were determined using the Micro Bicinchoninic Acid Protein Assay Protein Assay Kit (Thermo Fisher Scientific). BD-2 measurements, along with appropriate concentration adjustments, were performed as previously described for serum analyses and then expressed as a ratio to the total protein concentrations.

The protocol for protein extraction from tape-strips was optimized using the method described by Clausen et al. (49). Each tape was sonicated for 15 minutes with lysis buffer, and the protein extract was transferred to another tape. This protocol was repeated until protein was collected from a total of four tapes. The current study confirmed that protein concentrations were comparable to Clausen et al. and the optimized protocol to use 4 tape-strips per sample yielded nearly four times more total protein from healthy skin tape strips (49).

A broad evaluation of protein expression was also conducted on these lysates, using the Olink® Explore HT platform (Olink Proteomics) per manufacturer's protocol.

Skin biopsy processing for bulk RNA-sequencing

RNA lysates from skin biopsies were extracted using the Qiagen RNeasy Fibrous Tissue Mini Kit as per manufacturer's protocol. RNA Libraries were prepared from ~300 ng of total RNA using the KAPA RNA HyperPrep Kit with RiboErase (HMR; Roche) according to manufacturer's instructions. Sequence-ready libraries were validated and quantitated using the Agilent High Sensitivity D1000 ScreenTape Kit on the TapeStation 4200 System (Agilent Technologies). Individual libraries were normalized and quantified by qPCR using the KAPA Library Quantification Kit – Illumina/ROX Low (Roche) on the QuantStudio 12K Flex Real-Time PCR System (Applied Biosystems) according to manufacturer's instructions. The libraries were then pooled and quantified again by qPCR as described above. The quantified library pool was denatured and diluted according to the NovaSeq 6000 Denature and Dilute Libraries Guide and loaded onto a NovaSeq Reagent Kit v1.5 (200 cycles) for paired end sequencing using the NovaSeq 6000 System (Illumina). Binary base call (BCL) conversion and demultiplexing was performed using the bcl2fastq2 script (v2.20, Illumina).

Computational and statistical analyses

Participant and disease characteristic differences in baseline characteristics

Participant and disease characteristics at baseline were compared between placebo and combined icotrokinra participants with available serum samples using 1-way ANOVA for continuous

variables and Chi-squared for categorical variables. Due to small sample sizes, comparisons between placebo and 100 mg BID icotrokinra participants with available Olink tape-strip data were performed using Kruskal Wallis for continuous variables and Chi-squared for categorial variables, except baseline IGA 3/4, whose p-value was calculated using Fisher's Exact test.

PD Treatment effect on biomarker levels in serum and skin

Log_2 fold changes (log_2 FC) from baseline for seurm biomakers were measured using tracked estimated marginal means through Week 52. Comparisons between all icotrokinra doses and placebo or with baseline were performed at each time point from Week 4 to Week 16 using linear mixed effect models adjusted for the interaction of treatment with time and participant random effect. Error bars represent model-based 95% confidence intervals (CIs), and all p-values are nominal. Comparisons of BD-2 protein levels (µg/mL) between lesional and non-lesional skin and between Week 0 and Week 16 were performed using an unpaired t-test.

Correlation of biomarker levels and disease activity

Data from Weeks 0-16 for all participants were grouped into 6 PASI percent improvement categories: <25%, 25-49%, 50-74%, 75-89%, 90-99%, and 100%. BD-2 and IL-22 log_2 FC from baseline were compared across all PASI percent improvement categories; adjusted p-values were generated using Tukey multiple comparisons of means. Correlation between PASI change from baseline and biomarker change from baseline was performed using Pearson correlation.

Olink proteomic analysis

Differential protein analyte analysis was performed using the R package OlinkAnalyze (DOI: 10.32614/CRAN.package.OlinkAnalyze). Statistical testing was performed using the olink_ttest function from this package, which applied a Welch 2-sample t-test or paired t-test at confidence level 0.95 for every protein for a given grouping variable, correcting for multiple testing using the Benjamini-Hochberg method. Volcano plots for both serum and tape-strip samples were generated in R using ggplot2 with intensity-normalized protein expression (NPX) values. GSVA analysis was also performed on Olink proteomic data from tape-strip samples by generating protein-sets of the gene sets mentioned under the 'skin transcriptomic analysis' section below.

Skin transcriptomic analysis

Quality control of FASTQ files with raw sequence data was done using MultiQC tool (MultiQC is a quality control tool for high throughput sequence data. Available online at: https://multiqc.info/). High-quality reads were mapped to the human genome (GRCh38) using STAR/RSEM (52) with the in-house Green Beans pipeline. Read count matrices at the individual gene level were generated using kallisto (53). RNA-seq data analysis and GSVA were performed with the R package GSVA (50). Gene sets used for GSVA were from published data (39,41-43), including the previously described MAD-5 and MAD-3 gene sets (which contain genes that are differentially expressed between lesional and non-lesional psoriasis samples across 5 and 3 studies, respectively (39)), BioCarta pathways, MSigDB hallmark pathways, and internal Johnson & Johnson datasets. Unsupervised clustering of gene set enrichment z-scores in lesional and non-lesional skin biopsy samples was performed. Heatmaps of GSVA results were produced

using the pheatmap R package. Given the limited number of individuals participating only pooled icotrokinra versus placebo analysis was performed.

Population Pharmacokinetic and pharmacodynamic modeling

Data from all FRONTIER-1 and -2 participants were included in this analysis. The population pharmacokinetics (PK) was described using a one-compartment model with first-order absorption, linear elimination, and standard allometric relationship for the effect of body weight on apparent volume and clearance. A sequential PK/pharmacodynamic (PD) modeling approach was applied. Indirect response models involving a simultaneous fit of $\log_2$ BD-2 levels and PASI scores were used to describe the longitudinal population PK/PD of the PASI and $\log_2$ BD-2. The model for the drug effect on continuous PASI accounted for a placebo effect, as placebo data were included in the dataset. Each indirect response model had its own baseline and maximum effects, as well as different values for the first order elimination rate (k_{out}). Each response had its own residual error term. Random effects for both baseline responses and the maximum effect for the biomarker were included. Modeling and simulation of 1000 replicates for the VPC were performed using NONMEM (version 7.4).

Supplemental Tables

Table S1. Baseline demographic and disease characteristics for participants in the FRONTIER-1 study with tape-strip samples

**Skin tape-strip**	**Placebo** (N=29)	**100 mg BID** (N=31)	**P-value**
**Participant demographics**			
Age ,years	46.3 (13.6)	41.5 (11.6)	0.1489
Female/Male, %	55.2/44.8	35.5/64.5	0.2033
White/Asian, %	82.8/17.2	71.0/25.8	0.4243
Weight, kg	90.7 (27.2)	86.3 (17.2)	0.8591
**Psoriasis characteristics**			
Psoriasis duration, years	18.1 (15.2)	15.9 (12.5)	0.7504
PASI (0-72)	18.2 (4.5)	19.8 (6.6)	0.6149
BSA with psoriasis, %	25.0 (13.4)	22.0 (11.3)	0.3172
IGA, %			0.0817[a]
Severe (4)	6.9	25.8	
Moderate (3)	93.1	74.2	
**Previous psoriasis medications/therapies, %**			
Phototherapy[b]	48.3	35.5	NA
Biologics[c]	20.7	25.8	NA
Systemic[d]	86.2	87.1	NA

Data shown are mean (SD), unless otherwise indicated. [a]Includes participants with IGA 3 or 4. [b]Includes PUVA or UVB. [c]Includes etanercept, infliximab, adalimumab, ustekinumab, briakinumab, secukinumab, ixekizumab, brodalumab, guselkumab, risankizumab, tildrakizumab, alefacept, efalizumab, natalizumab, certolizumab pegol. [d]Includes conventional nonbiologic systemic therapies, novel nonbiologic systemic therapies, 1,25-vitamin D3 and analogues, phototherapy, and biologics. BID: twice daily; BSA: body surface area; IGA: Investigator's Global Assessment; PASI: Psoriasis Area Severity Index; PUVA: psoralen plus ultraviolet A; UVB: ultraviolet B.

Supplemental Figures

Figure S1. Olink Explore HT protein quantification of Week 0 lesional psoriatic skin versus control skin from healthy volunteers.

Log_2 FC = 1 or -1 and adjusted p-value = 0.05 thresholds are depicted with dotted lines. Enrichment statistics were based on Benjamini-Hochberg procedure. [a]Healthy control skin tape-strip samples were obtained from sponsor healthy donor program at Johnson & Johnson (Protocol NOCOMPOUNDNAP1001).

BD: beta defensin; IL: interleukin; ns: not significant; PI: peptidase inhibitor.

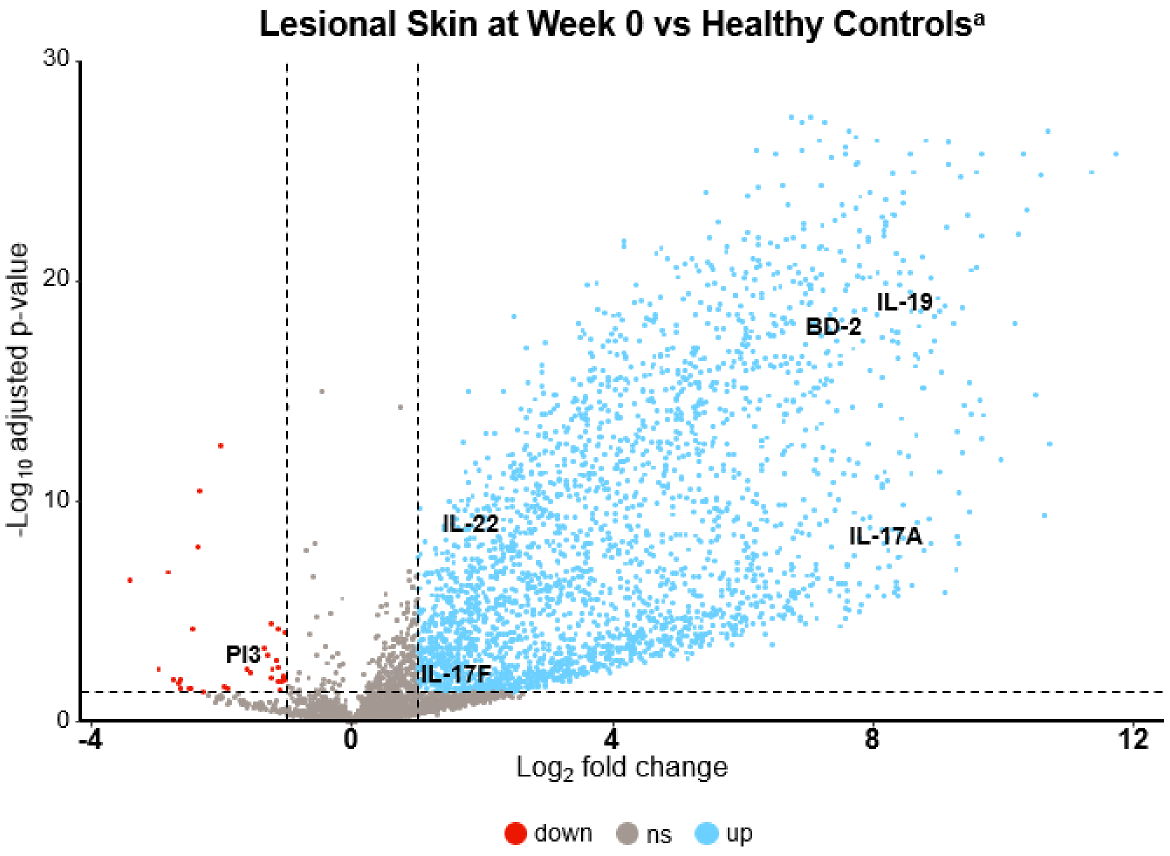

Conflict of interest: DS, D Richards, EYC, KP, DH, RP, JJR, JS, SB, LPT, CHC, MEP, EB, YX, WZ, CD, PN, DMW, MM, TO, YWY, MWLL, LSM, CAC, BM, D Ruane, AKK: Shareholder: may own stock/stock options in Johnson & Johnson. JGK: Consultant/honoraria: AbbVie, Aclaris, Allergan, Almirall, Amgen, Arena, Aristea, Asana, Aurigene, Biogen, Boehringer Ingelheim, Bristol-Myers Squibb, Eli Lilly, Escalier, Galapagos, Johnson & Johnson, MoonLake, Nimbus, Novartis, Pfizer, Sanofi, Sienna, Sun, Target-Derm, UCB, Valeant, and Ventyx. RB: Advisory Board Member, Consultant, Speaker and/or Investigator for and received honoraria and/or grants from AbbVie, Alumis, Amgen, Arcutis, Bausch Health, BMS/Celgene, Boston Pharma, Dermavant, Eli Lilly, Johnson & Johnson, LEO Pharma, Nimbus, Novartis, Pfizer, Regeneron, UCB, VentyxBio, Vyne, Xencor and Zai Lab. R Bissonnette is also an employee and shareholder of Innovaderm Research. KE: Advisory board member and/or speaker: AbbVie, Almirall, Boehringer Ingelheim, Bristol-Myers Squibb, Eli Lilly, Hexal, Johnson & Johnson, LEO Pharma, Novartis, Pfizer, Sanofi, Sitryx, and UCB; Co-founder/shareholder: Dermagnostix and Dermagnostix R&D. LKF: Investigator: AbbVie, Acelyrin, Amgen, Apogee, Arcutis, Aristea, Boehringer-Ingelheim, Bristol-Myers Squibb, Cara Therapeutics, Castle Biosciences, DermTech, Eli Lilly, Galderma, GRAIL, Incyte, Johnson & Johnson, LEO Pharma, Moberg, Mobius, Novartis, Regeneron, SkinAnalytics, Takeda, and UCB; Consultant: AbbVie, Amgen, Apogee, Arcutis, Boehringer-Ingelheim, Bristol-Myers Squibb, Cara Therapeutics, Dermavant, DermTech, Johnson & Johnson, LEO Pharma, Novartis, Pfizer, Regeneron, and Takeda; Speaker: AbbVie, Arcutis, Boehringer-Ingelheim, Bristol-Myers Squibb, and Regeneron. ASP: Investigator: AbbVie, Biomendics, Dermavant, Eli Lilly, Incyte, Johnson & Johnson, Regeneron, UCB. Consultant: Abeona, Arcutis, BioCryst, Boehringer-Ingelheim, Castle Creek, Chiesi, Dermavant, Johnson & Johnson, Krystal, LEO Pharma, Eli Lilly, L'Oreal, MoonLake Immunotherapeutics, Peltheos, Quoin, Regeneron, and Sanofi; Data safety monitoring board: AbbVie, Abeona, Biocryst, Daiichi Sankyo, and Galderma. AP: Advisor, grants, investigator, and/or speaker: AbbVie, Almirall Hermal, Amgen, Biogen Idec, BioNTech,

Boehringer Ingelheim, Celgene, Celltrion, Eli Lilly, Galderma, GlaxoSmithKline, Hexal, Johnson & Johnson, Klinge Pharma, LEO Pharma, MC2, Medac, Merck Serono, Mitsubishi, MSD, Novartis, Pascoe, Pfizer, Regeneron, Roche, Sandoz Biopharmaceuticals, Sanofi Genzyme, Schering-Plough, Tigercat Pharma, and UCB Pharma, and Zuellig Pharma.
BEK:Former Employee: Johnson & Johnson; Shareholder: stock/stock options in Johnson & Johnson; Employee: Mirador Therapeutics.
